# Test strategy for assessing the risks of nanomaterials in the environment considering general regulatory procedures

**DOI:** 10.1186/s12302-015-0053-6

**Published:** 2015-10-06

**Authors:** Kerstin Hund-Rinke, Monika Herrchen, Karsten Schlich, Kathrin Schwirn, Doris Völker

**Affiliations:** 1 Fraunhofer Institute for Molecular Biology and Applied Ecology IME, Auf dem Aberg 1, 57392 Schmallenberg, Germany; 2 German Federal Environment Agency (UBA), Wörlitzer Platz 1, 06844 Dessau, Germany

**Keywords:** Assessment, Test strategy, Nanomaterials, Ecotoxicology, Fate, Environment

## Abstract

**Background:**

Engineered nanomaterials (ENMs) are marketed as a substance or mixtures and are additionally used due to their active agent properties in products such as pesticides or biocides, for which specific regulations apply. Currently, there are no specific testing strategies for environmental fate and effects of ENMs within the different regulations. An environmental test and risk assessment strategy for ENMs have been developed considering the general principles of chemical assessment.

**Results:**

The test strategy has been developed based on the knowledge of national and international discussions. It also takes into account the conclusions made by the OECD WPMN which held an expert meeting in January 2013. For the test strategy development, both conventional and alternative endpoints were discussed and environmental fate and effects were addressed separately.

**Conclusion:**

A tiered scheme as commonly used in the context of precautionary environmental risk assessment was suggested including the use of mathematical models and trigger values to either stop the procedure or proceed to the next tier. There are still several gaps which have to be filled, especially with respect to fate, to develop the test strategy further. The test strategy features a general approach. It is not specified to fulfil the information requirements of certain legislation (e.g. plant protection act, biocide regulation, REACH). However, the adaption of single elements of the strategy to the specific needs of certain legislation will provide a valuable contribution in relation to the testing of nanomaterials.

## Background

Engineered nanomaterials (ENMs) are marketed as a substance or mixtures and additionally used due to their active agent properties in products like, e.g. pesticides or biocides, for which substance and product specific regulations apply [1, 2]. Currently, there are no nano-specific information requirements for environmental fate and effects of ENMs within the different regulations. In several projects under the EU’s Seventh Framework Programme for Research (FP7), test strategies to assess human and environmental risk are discussed and the topics to be further investigated listed. An excellent example is the project ITS-NANO (ITS: Intelligent Testing Strategy) which has delivered a detailed, stakeholder driven and flexible research prioritization (or strategy) tool, which identifies specific research needs, suggests connections between areas, and frames this in a time perspective [3]. In projects as MARINA or SUN, tools and strategies for a risk assessment of manufactured nanomaterials are being developed. Usually, the established procedure for testing and risk assessment of conventional chemicals is taken into account in these projects. Additional endpoints (such as biomarkers), test systems (such as more generation tests), and multiple application as well as ageing of ENMs in the respective test medium are discussed. The EU NanoSafety Cluster was initiated by the DG RTD NMP to maximise the synergies between the EU projects addressing all aspects of nanosafety including toxicology, ecotoxicology, exposure assessment, mechanisms of interaction, risk assessment and standardisation (http://www.nanosafetycluster.eu/).

**Fig. 1:**
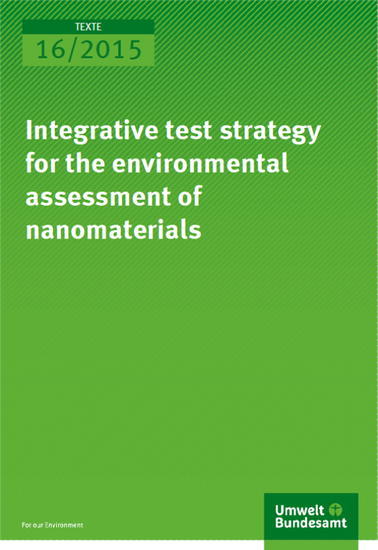
Cover of the report supported by the Federal Environment Agency (Umweltbundesamt). The entire report is available at http://www.umweltbundesamt.de/publikationen/integrative-test-strategy-for-the-environmental

The aim of this study was to develop a test and risk assessment strategy for ENMs which specifically addresses environmental fate and effects (Fig. 1). For both of these, precise test systems and strategies of data collection, and evaluation are provided. To our knowledge, the level of detail and comprehensiveness, and the resulting recommendations exceed published approaches.

The test strategy has been developed based on a literature review and on the knowledge of national and international discussions, after comparison with the proposals presented by the Reach Implementation Project on Nanomaterials 2 (RIP-oN) and by German competent authorities for REACH (regulation concerning the registration, evaluation, authorisation and restriction of chemicals) and CLP (regulation on classification, labelling and packaging of substances and mixtures). It also takes into account the conclusions agreed at the OECD WPMN (OECD working party on manufactured nanomaterials) which held an expert meeting in January 2013 on the suitability of test guidelines for environmental fate and ecotoxicity [4]. The literature review was performed in 2012, with additions in 2013 and 2014 with the aim to present an overview of the state of the art. It was not intended to provide a compilation of all available references. In the following, the main steps of the test strategy and results which emerged from this conceptual work are presented. The entire report is available at http://www.umweltbundesamt.de/publikationen/integrative-test-strategy-for-the-environmental.

## Results and discussion

### Overview on the test strategy

**Fig. 2:**
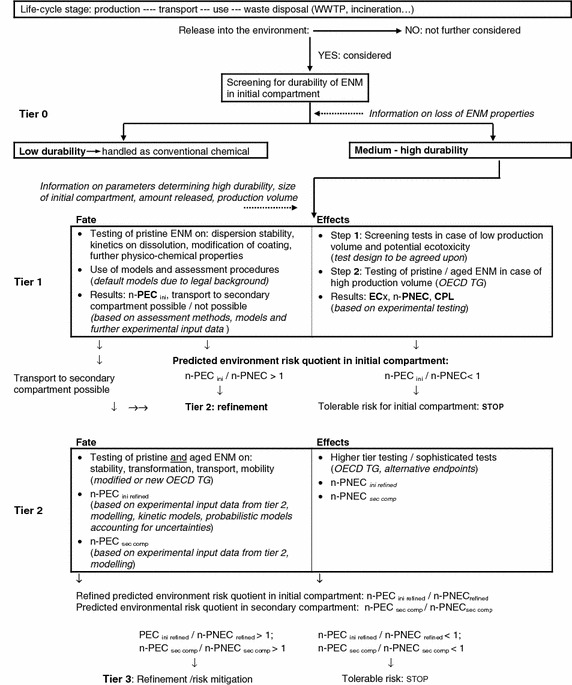
Overall test strategy including fate and ecotoxicity

The presented approach (Fig. 2) is a life-cycle oriented one, and thus considers all stages along the life of the ENMs. In particular, these are: production, transport and distribution to the user, use, and waste management. Further, transport stages might occur, e.g. the transport of the used ENMs to an incineration plant. For each single stage, it has to be considered whether there is a potential for the ENMs to be released into the environment. Furthermore, with respect to each single stage, the initial environmental compartment in which the ENMs are expected to be released into has to be identified. In the test strategy, we consider the compartments: water, sediment and soil. If the release potential is negligible, this particular life-cycle stage needs no further consideration. It has to be noted that the definition of “negligible” and “non-negligible” with regard to this test strategy has still to be discussed.

At the beginning, the durability of the tested ENMs in the initial compartment should be screened (tier 0). The term “durability” means that the ENMs keep their status as a nanomaterial. For that screening, any information about the ENM properties and their possible loss (e.g. by rapid dissolution) is indispensable. Such information should be available (at least to some extent), e.g. from the manufacturer collecting it in the course of product design and development. In the case, that low durability is determined, meaning that the ENM rapidly loses its status of being a nanomaterial, the formed chemicals can be tested and assessed as conventional chemicals. In the case that medium to high durability is determined, the first tier of the assessment scheme is initiated. It has to be noted that any trigger value to differentiate between “high”, “medium” and “low” durability has still to be discussed. We expect that for example metals and metal oxides will belong to the medium and high durability group, whereas most of the “nano pesticides” will be allocated to the low durability group.

For tier 1, both a fate and effect assessment have to be performed. The assessments result in a predicted environmental concentration in the initial compartment (n-PEC_ini_) and a predicted no effect concentration (n-PNEC). The prefix “n” is used to characterise the PEC and PNEC as concentrations for “nanomaterials”.

The deduction of n-PEC _ini_ needs the information on experimental physico-chemical characteristics as well as preliminary data on environmental behaviour of the ENMs, i.e. information on the agglomeration behaviour, stability of the coating, and alteration of the ENMs, e.g. by oxidation or dissolution. For information on some of these endpoints, modified or even newly developed test guidelines and guidances, e.g. on agglomeration behaviour or dissolution rate, will be necessary. The deduction of n-PEC_ini_ also needs information on the production volume as well as on the amount of the ENMs released in every life-cycle stage. Furthermore, it requires a specification of the volume of the initial compartment, e.g. the definition of a local or regional scenario. Finally, the definition of default models which is already applied for the exposure assessment of conventional chemicals, plant protection products and biocides is also considered for the derivation of n-PEC_ini_.

The effect assessment resulting in n-PNEC for low production volume nanomaterials, which have non-toxic non-nano counterparts, is determined by screening tests representing the respective initial compartment (named as “step 1” in the scheme). For all other ENMs, OECD test guidelines suitable for the testing of ENMs are used (named as “step 2” in the scheme). Thus, the effect testing at tier 1 comprises two different levels of complexity. It has to be noted that trigger values for “low” and “high” production volume still have to be discussed. Furthermore, the test design of the screening tests needs mutual consent. NOEC values or ECx values are the outcomes of any of the experimental testing. Using assessment factors/uncertainty factors, well-known from the risk assessment of conventional chemicals, a predicted no effect concentration (n-PNEC) can be derived. Besides n-PNEC values, a classification and product labelling (CPL) on the basis of the effect concentrations is conceivable.

Comparable to conventional chemicals, a risk quotient (n-PEC_ini_/n-PNEC) can be derived. In case it is below 1, a tolerable risk for the initial compartment can be assumed. No further sophisticated risk assessment for the initial compartment is needed. In case it is above 1, the risk for the initial compartment might not be negligible and, thus, a refinement at tier 2 is needed.

Regardless of the risk quotient for the initial compartment, a possible ENM transport to a secondary compartment—e.g. the transport from the aqueous phase to the sediment and transport within the sediment—needs further consideration. The transport potential will be assessed on the basis of physico-chemical data, the preliminary tests on environmental behaviour, size, and size distribution rather than on complex fate tests. If transport to a secondary compartment is expected, this compartment has also to be addressed by a risk assessment at tier 2.

The refined n-PEC-assessment at tier 2 comprises two aspects: on the one hand, a refinement for the initial compartment (n-PEC_ini refined_), and on the other hand an assessment for a second compartment if the ENM might be transported into it (n-PEC_sec comp_). Both need experimental input data and modelling. The experimental fate testing at tier 2 will also need data from modified or even newly developed test guidelines and guidances. Furthermore, testing at tier 2 will also consider environmental behaviour of ENMs altered in the test system. n-PEC_ini refined_ and n-PEC_sec comp_ are assessed by the use of kinetic models. That accounts for the fact that environmental fate processes of ENMs are kinetic processes but not equilibrium processes as they are for conventional chemicals [5]. Furthermore, it is advisable, at least on the current state of knowledge, to use probabilistic models in order to account for the uncertainties of the model input parameters.

The refined n-PNEC-assessment at tier 2 comprises a higher tier testing, i.e. the use of more sophisticated tests such as water/sediment studies or aquatic mesocosm/terrestrial microcosm studies or the use of alternative endpoints whose appropriateness for utilisation in a refined PNEC assessment still needs to be investigated. In the case of a likely exposure of a secondary compartment, appropriate effect tests have to be performed. The testing and the use of assessment factors result in n-PNEC_refined_.

Tier 2 yields a refined risk quotient for the initial compartment (n-PEC_ini refined_/n-PNEC_refined_) and, in the case of a likely exposure of a secondary compartment, in a risk quotient for that compartment (n-PEC_sec comp_/n-PNEC_sec comp_). As at tier 1, the trigger of 1 is used to either “STOP” or to proceed to a further tier. Tier 3 might comprise an additional even more sophisticated test refinement or measures for risk mitigation.

### Effect assessment

**Fig. 3:**
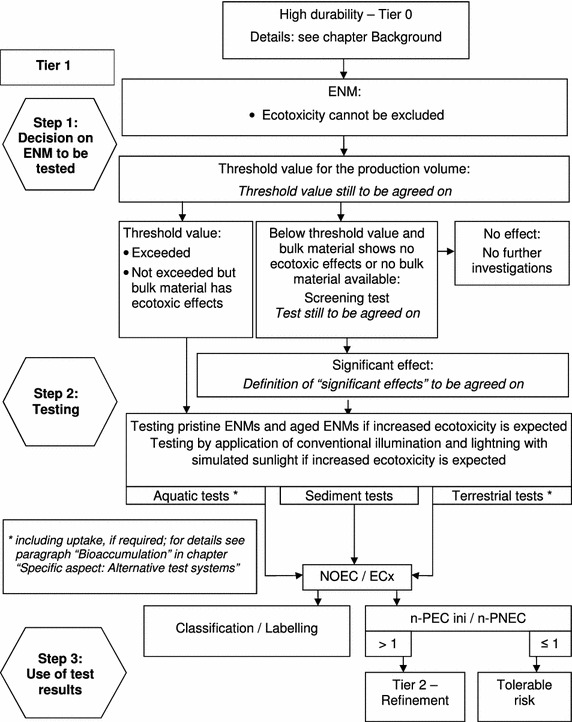
Basic test strategy for the ecotoxicological testing of ENMs (*Tier 1*)

The basic test strategy on effect assessment comprises three phases: (I) decision on the ENMs to be tested, (II) comprehensive testing and (III) use of test results (Fig. 3).

#### General aspects

##### ENMs to be tested

In the first step of the test strategy, it has to be decided whether or not the nanospecific test procedures presented here have to be followed. Under following two conditions, it appears acceptable to waive testing:

- ENMs featuring physicochemical parameters well known for indicating nontoxic potential in every environmental compartment (water, sediment, soil) without any doubt.
- There is proof that a direct or indirect exposure of the environment can be excluded.

For regulatory testing, the decision on waiving of testing can be proposed by the registrant and has to be justified. The final decision on acceptance of the waiving is decided by the regulatory body. For chemical substances under REACH information requirement is triggered by their production volume. Four different ranges of production volumes per year are agreed on for which ecotoxicity data are required: 1 to <10 t/a; 10 to <100 t/a; 100 to <1000 t/a; 1000 t/a and more). Chemicals with a high production volume have to be tested more comprehensively than chemicals with a lower production volume. For chemicals with low production volumes, only aquatic tests are requested whereas for chemicals with high production volume additionally terrestrial and sediment tests are required. In the presented test strategy for ENMs, only one threshold value based on one, rather low production volume is intended. This value still has to be defined. ENMs with production volumes exceeding this threshold value have to be comprehensively tested addressing endpoints in the three compartments: water, sediment and soil. Also ENMs with a lower production volume than the threshold can be toxic to the environment. Therefore, two scenarios have to be considered. For low volume ENMs with bulk material of known ecotoxicity, comprehensive investigation has to be performed. For low volume ENMs where no bulk material is available, the bulk material shows no ecotoxicity, or ecotoxicity of the ENMs is expected due to their physico-chemical properties, a screening test has to be performed. If toxic effects are detected in the screening test, comprehensive testing has to be performed.

In summary, according to the presented test strategy testing is requested under the following conditions:

Comprehensive testing for:

- ENMs with production volume above the threshold value
- ENMs with production volumes below the threshold value and ecotoxic bulk material
- ENMs with production volumes below the threshold value and effects seen on the screening test

Screening test for:

- ENMs with production volume below the threshold value and bulk material is not ecotoxic or no bulk material is available or ecotoxic effect expected based on physical–chemical properties

If ENMs, due to their application, are covered by specific legislation where production volumes are not considered (e.g. ENMs used as biocides or pesticides), the aspect “production volume” is neglected in the presented test strategy. Instead, these ENMs are investigated starting with step 2 of tier 1 in order to comply with the requirements of the respective legislation, but also to consider nanospecific aspects as proposed within this test strategy (e.g. selection of tests: chronic tests instead of acute tests).

##### Screening test

The screening test and the interpretation of the results still have to be discussed. The screening test should be used as a tool for identifying hazardous ENMs of low production volume whose corresponding non-nano counterparts give no hint on ecotoxicological potentials. Therefore, the test needs to have high sensitivity; the indicator function concerning effects on populations is less important in this case. The criteria for such tests should be (1) easy to perform and low work load, (2) short test duration, (3) sensitivity comparable to the sensitivity of the standardised endpoints to avoid too many “false” positive or “false” negative results. In several publications, high-throughput assays are described [6, 7]. Besides testing, also modelling might be a useful alternative. By the use of models toxic properties of ENMs such as oxidative stress potential of oxide, ENMs may be predictable [8, 9]. The suitability of such methods and procedures for the initial examination has to be investigated and the most reliable procedures need to be further developed. If the results indicate considerable toxicity, these ENMs enter step 2 of tier 1 (comprehensive testing).

##### Comprehensive testing

Comprehensive testing is required for ENMs with production volumes exceeding the threshold value, for low volume ENMs which show toxicity in the screening test, and for ENMs which are covered by specific legislations where production volumes are not considered.

So far, there are considerable knowledge gaps with regard to the sensitivity of aquatic tests in comparison to terrestrial tests. It cannot be excluded that terrestrial tests are of comparable sensitivity or even more sensitive than aquatic tests as differences in the exposure concentration of the investigated ENMs between soil and aquatic tests are expected. In aquatic tests, the exposure and availability can change due to agglomeration and, depending on the test conditions, sedimentation. Sedimentation will result in increased exposure for sediment organisms. In contrast, in soil the exposure concentration of the organisms is not expected to change dramatically based on agglomeration. Therefore, the situation of exposure and availability and its changes are expected to be considerable differences between these three compartments and thus the test strategy includes a test programme which considers all three compartments, namely surface water, sediment, and soil.

Since it is assumed that ENMs preferentially enter the sediment compartment via the water phase, a test on sediment organisms performed using spiked water seems more appropriate to simulate the primary exposure scenario. Due to movement of the sediment organisms, sedimented ENMs can be incorporated into the sediment and spiking of sediment simulates the secondary exposure scenario. ENMs can be subjected to alterations of their physical–chemical characteristics in environmental media over time which in turn influences behaviour, bioavailability and toxicity. Furthermore, biodegradability of most ENMs is limited due to their inorganic condition and persistence is expected. Therefore, tests with longer incubation periods are preferred. Regarding the standardised test systems, the following test programme for ENMs is considered for the test strategy:

- Aquatic tests
  Daphnids: OECD TG (test guideline) 211 [10]; algae: OECD TG 201 [11]; fish: OECD TG 210 “Fish, Early-life Stage Toxicity Test” [12]
- Sediment test
  Chironomids: OECD 218, 219 [13, 14] (spiked sediment and spiked water phase) or lumbriculus: OECD TG 225 [15] (so far, a TG for the Lumbriculus test using spiked water is not available and a development is recommended)
- Terrestrial tests
  Microflora: OECD TG 216 [16] using an inorganic nitrogen source instead of an organic one; earthworms: OECD TG 222 [17]; plants: OECD TG 208 [18]
  (Explanation for inorganic nitrogen source in a test according to OECD TG 216 [16]: Based on a recent study, it is anticipated that released ions tend to sorb to the additional organic nitrogen source, thus reducing their bioavailability. As a consequence, the use of an inorganic nitrogen source or a test on potential ammonium oxidation according to ISO Guideline 15685 [19, 20] can resolve this limitation.)

In this context, the quality control of the experiments and the validity of the test results have to be emphasised as addressed by Rösslein et al. [21] for tests in microtiter plates. Subjects such as homogeneity of spiking, sedimentation and concentration of ENMs over time, reactions with components of the test media and photoreactivity of the ENMs have to be considered and appropriate controls for ecotoxicological tests with organisms and complex test designs have to be established.

The need for revising current OECD test guidelines and the development of new ones was discussed by experts from science, industry and regulatory bodies at an OECD workshop on ecotoxicology and environmental fate of ENMs in 2013. An overview on the discussions and recommendations is given in Kühnel and Nickel [4]. The main subjects which have to be considered in the adaptation of the ecotoxicological test guidelines are spiking of terrestrial and aquatic test systems and the exposure of organisms in aquatic systems. For ENMs available as a powder, it has to be decided whether application via stock suspension (wet application) or via powder (dry application) is recommended. For aquatic tests, important issues were discussed at a workshop and recently published [22].

The metric to be used for the calculation of the toxicity is still being discussed. Besides mass, also size/surface area of ENMs and particle number may be suitable. To allow comparability with the results obtained with conventional substances, results should be presented on a mass basis. In addition, physical–chemical characterisation of the ENMs and the methods used for the determination should be reported. If required, the results can be recalculated using the metric “surface area” or “particle number”. However, the reliability of a recalculation depends on the available information on particle size distribution of the respective ENM).

In several terrestrial and aquatic tests with various ENMs, a plateau with a maximum effect below 100 % is observed instead of concentration–effect relationships with a maximum of 100 % effect [23, 24]. The background of these observations is not yet systematically investigated but it is assumed that limitations in bioavailability and exposure are responsible. Therefore, a limit test with several test concentrations instead of only one test concentration is preferred to obtain information about the dose–response relationship.

The test conditions described in the test guidelines usually do not support photocatalytic activity. Simulated sunlight can increase ecotoxicity of photocatalytic active ENMs and possibly also of further ENM types [25, 26]. Aquatic tests should be performed according to the guidelines and additionally with simulated sunlight for photocatalytic ENMs. The tests with conventional lighting are recommended to address the unspecific properties of these ENMs in the absence of photoinduction and to link the results to results obtained by applying the test guidelines. The most sensitive result, independent of the illumination conditions, should be used for the assessment of hazard of ENMs. In addition, knowledge has to be improved with respect to illumination-dependent ecotoxicity of ENMs which are not specifically designed to feature photocatalytic activity but whose properties or behaviour are influenced by illumination [27].

##### Use of test results

The test results can be used to describe the ecotoxicological properties of ENMs. Additionally, classification and labelling as well as an initial environmental hazard and risk assessment can be performed. For each purpose, only the relevant test results, as required in the respective regulation, need to be used. With respect to ecotoxicity, classification and labelling should address the most endangered environmental compartment. Currently, only the aquatic compartment is considered in classification and labelling and guidance for the other compartments has to be developed if required.

For the characterisation of the hazard with respect to risk assessment, PNEC values are required. For conventional chemicals, uncertainty in hazard can be considered using assessment factors [28]. Currently, there are no indications that assessment factors differing from the existing ones are needed for ENMs. For risk assessment, the PNEC values have to be compared with environmental concentrations (PEC). The topic of risk assessment is addressed in the chapters “Overview on the test strategy” and “Risk assessment approaches”.

#### Specific aspect: alternative test systems in the test strategy for the assessment of ENMs

The appropriateness of the OECD test guidelines as well as other guidelines for nanomaterials has been reviewed and it is generally accepted that most endpoints are adequate and relevant also for ENMs [29]. Some modifications of the test procedures are required [4] and currently for some of the OECD test guidelines nanospecific guidance is drafted as well as some new OECD test guidelines are being developed.

Besides the application of the standardised test methods, alternative test methods and endpoints for the assessment of ENMs are published. So far, it is not clarified whether these endpoints provide additional information within the framework of regulation justifying the integration in the test strategy for ENMs. A literature review on alternative test methods such as behaviour, nutritional performance, indicator for oxygen stress, haematology, histology, genotoxicity, cytotoxicity, neurotoxicity, immunotoxicity, bioaccumulation and biodiversity was performed in the project and the following conclusions were drawn:

1. The conventional endpoints used for hazard assessment are selected with respect to the protection of populations and cover parameters such as reproduction, mortality, growth. Effects on individuals are not considered. The results on alternative parameters reviewed in the literature usually address less complex and sub lethal reactions (e.g. determination of specific enzymes or gene activities) at a level of a single or some individuals, often resulting in an increased sensitivity. It is not always obvious whether an effect detected by a sensitive additional endpoint (e.g. indicators for oxygen stress) has an impact on the population level or indicates a compensation measure of the organism. Based on the literature review, it can be concluded that the advantage of considering alternative endpoints as additional input for a regulatory hazard assessment specific for ENMs is limited so far. Nevertheless, every additional parameter can provide additional information on ecotoxicity of ENMs and can support the assessment. In any case, in research, alternative endpoints play a major role by increasing the knowledge on the mode of action of ENMs.
2. There are some specific effects which are not detected with the conventional endpoints but which might have an impact on the population level and as such might be of relevance for assessing the hazard of ENMs for regulatory purposes.
  - Immunotoxicity/genotoxicity
    The knowledge of the significance of effects on immunotoxicity and genotoxicity caused by ENMs in vitro and on the population level should be improved. Furthermore, the results have to be compared with the results obtained within the scope of studies on human toxicology. Based on this information, it can be decided whether these parameters are a suitable addition to the ecotoxicological test strategy.
  - Bioaccumulation
    Bioaccumulation is actually considered for fate and behaviour aspects. However, in the present study, it was taken into consideration as an alternative endpoint delivering additional information on ecotoxicity. So far, the knowledge on physicochemical parameters indicating accumulation of ENMs is limited. Generally, the determination of bioaccumulation needs to account for the fact that uptake and distribution processes of ENMs are kinetically driven. Thus, to obtain initial information on the accumulation potential and uptake of ENMs, a pragmatic screening procedure is to determine the ENM concentration in suitable test organisms (e.g. terrestrial and aquatic oligochaetes, daphnids, fish embryos and plants) at the end of the incubation period in an ecotoxicological test. If more detailed results are required, specific studies on bioaccumulation can be performed taking into account the discussions of the OECD expert meeting [4] and, once available, specific guidance on the accumulation of ENMs. Furthermore, this screening procedure for accumulation can be used to identify physicochemical parameters indicating bioaccumulation.
  - Multi-generation tests
    It can be assumed that the effects become more pronounced if multi-generation tests are performed. Additionally, recovery studies can provide relevant information [30]. Even though the experimental effort is quite high, the consideration of multi-generation tests and recovery studies may result in a higher significance of the hazard assessment. However, uncertainty with respect to the significance of the assessment based on data of conventionally applied acute and chronic toxicity tests is considered, e.g. by assessment factors. Furthermore, it is assumed that multi-generation tests and recovery studies feature additional information specific not only for ENMs but also for conventional chemicals. There is no reason to consider such test approaches for only one group of chemicals. Nevertheless, knowledge on long-term effects should be improved to adapt the test strategy if necessary.
  - Further test organisms
    ENMs agglomerate in aquatic systems, and increased concentrations in the sediment are expected [31]. The standardised test organisms *Chironomus riparius* and *Lumbriculus variegatus* develop in the sediment. It cannot be excluded that organisms living and grazing on the sediment as well as floated submerged, aquatic macrophytes are exposed to a higher extent compared to the standard test organisms if spiking of the water phase is performed. It is recommended that the sensitivity of potential suitable organisms (sediment organisms, aquatic macrophytes) and of the standard test organisms (*C.riparius*, *L. variegatus*, *Lemna minor*) are compared to decide on the suitability of further test organisms and the potential replacement of traditionally applied organisms for the testing of ENMs.
  - Behavioural tests
    In the reviewed literature, behavioural tests appeared to be quite sensitive. However, so far, the information on the applicability on a wide range of ENMs is limited. To extend the knowledge, the behavioural test with earthworms [32] was studied in more detail in the experimental section of this project. It became obvious that the avoidance test with its short incubation period can provide important information on ecotoxicity and ageing of ENMs. However, a general utilisation within the test strategy for regulatory purposes, i.e. as a screening test, is not recommended since false-negative assessments cannot be excluded.

### Fate assessment

#### General

The basic test strategy for fate endpoints of ENMs needed for their exposure assessment comprises three tiers: (0) screening for durability of ENMs in the initial compartment and thus a decision on the ENMs to be tested, (I) tier 1 to determine n-PEC _ini_ and transport to secondary compartments (II) tier 2 to refine the results of tier 1. Tier 0 already has been presented in sufficient detail in the introduction and is not addressed furthermore. The determination of n-PEC_ini_, transport to other compartments and the PEC refinement are based on experimental fate data. Examples of needs and challenges in tier 1 and tier 2 testing are presented in the following chapters and the test strategy considers the environmental compartments water, sediment, and soil. For the development of the test strategy on fate, a comprehensive literature review was performed. The aim of this evaluation was to summarise and analyse endpoints on fate and behaviour as well as the corresponding test methods for their importance and appropriateness to be implemented into a test strategy on the fate of ENMs. The detailed literature evaluation including results on single ENMs investigated using specific test methods is available in the report (http://www.umweltbundesamt.de/publikationen/integrative-test-strategy-for-the-environmental).

#### Tier 1 testing

The n-PEC_ini_ is determined on the basis of information on the stability as a dispersion or emulsion, stability of the organic coating, and modification of the ENM, e.g. by oxidation, dissolution/solubility rate, size and size distribution. This information is also used to elucidate whether or not transport to secondary compartments is possible which triggers refinement in tier 2. In addition, default models currently applied for the different compartments are used to deduce the n-PEC_ini_.

Testing of fate endpoints of ENMs has to take into account that environmental fate processes of ENMs are mainly kinetically driven and include homo- and hetero-agglomeration (with suspended organic matter or biota) as well as transport processes like sedimentation in aquatic media [5]. Thus, it is commonly accepted [33–35] that guidelines which are based on partitioning processes are not suitable for ENMs, since employing partitioning coefficients to describe the behaviour of ENMs in the different environmental compartments will inevitably lead to misinterpretations of ENM distribution.

Since stability of ENMs in the environment is strongly influenced by the composition of the surrounding compartment, this indicates that for the determination of the n-PEC _ini_ interaction with media compartments has to be considered (e.g. pH, NOM, ionic strength) [36–39].

Stability in the sense of biodegradation is measured based on the oxidation of organic carbon (e.g. BOD determination). However, these tests are expected to be applicable in rare cases only since most of the known ENMs are of inorganic nature. Thus, alternative approaches are needed to describe the general transformation of ENMs in the environment. The presented test strategy suggests that these approaches are covered by endpoints like agglomeration, dissolution or transformation upon ageing. In addition, transformation of ENMs based on the biological, chemical or physical loss of the coatings needs to be considered.

Based on the literature evaluation and discussions of the scientific and regulatory communities [4], it became obvious that for some of the mentioned endpoints like agglomeration and dissolution new or modified test guidelines are needed (e.g. [40–44]).

#### Tier 2 testing

In the case of an n-PEC_ini_/n-PNEC-ratio of >1 risk for the considered compartment or transport to a secondary compartment cannot be excluded, a PEC refinement is needed. The refinement requires further experimental fate data as input for a more sophisticated modelling for exposure assessment. In the sense of the presented test strategy, most important endpoints to be considered include further (a) biotic transformation/degradation, mobility and transport in porous media, and sorption to soil, sediment and sludge of the pristine and aged ENM. The more complex the considered environmental matrix, the more environmental parameters interact with ENMs which themselves are of a complex nature. As a consequence thereof, the experimental test design needs to reflect this: the more complex the tested compartment, tier 2 considers experimental setups stronger mimicking the representative environmental compartment [45]. This can in particular be achieved using soil column experiments, e.g. as described in OECD 312 or by even more complex laboratory test systems such as model waste water treatment plants [46, 47] or fresh water mesocosms [33]. Most importantly, various techniques, in particular analytical techniques, should be combined to obtain a comprehensive and reliable picture of the ENM mobility, e.g. in porous media. It has to be considered that most experimental setups are likely to affect the form in which ENMs occur and might yield a result that is not representative of the behaviour under realistic environmental conditions. Furthermore, ENM properties like shape, crystal structure and surface properties influence mobility and transport as well as sorption/desorption to soil, sediment, and sludge and, therefore, have to be taken into account [48] within assessing fate in tier 2. It has to be noted that the concept of sorption is based on distribution coefficients and is of major importance for the description of solutes transport in soil. However, ENM association with soil is a non-equilibrium process, as it is also in other environmental compartments. Existing test methods appropriate for conventional chemicals, i.e. OECD TG 106, will generate misleading results [48, 49]. Alternative endpoints need to be employed to describe major processes influencing mobility and transport including agglomeration, deposition and re-mobilisation [48]. As already mentioned, for some of these endpoints new or modified test guidelines are needed.

#### PEC assessment, PEC models

Environmental fate processes of ENMs which are mostly influenced by aggregation, transformation and sedimentation are non-equilibrium but kinetic processes. ENMs do not reach thermodynamic equilibrium but are present in the environment as suspensions of different stability [5, 50]. Thus, conventional distribution models based on equilibrium processes such as the fugacity models developed by Mackay [51] are not applicable. ENM fate models have to be designed and evaluated which are capable of incorporating the environmental complexity to predict realistic environmental concentrations of ENMs. Furthermore, the use of kinetic models is essential in PEC assessment [4, 47].

Quite often reliable data are missing, e.g. on the quantity of emissions into the environment during production and usage. This situation can be overcome to some extent using probabilistic density functions [52].

### Risk assessment approaches

Hazard and fate data are two essential parts of the analysis of the environmental risk of ENMs. Only a few references of the literature review conducted in this study deal with the risk assessment of ENMs. These comprise:

- Comparison of risk assessment of conventional substances and risk assessment of ENMs.
- Dealing with uncertainties and limited input information.
- Integration of ENM alteration and transformation in the risk assessment

Uncertainties regarding the potential impacts and risks associated with ENMs were discussed by Adam [34]. The authors combined life-cycle assessment (LCA) and risk assessment approaches. Because high uncertainties remain concerning the fate and effects of ENMs probabilistic approaches are needed, a Bayesian network was used. Nowack et al. [53] concluded that the risk due to ENMs cannot be determined exclusively for pristine ENMs, but has to consider alterations and transformation in the environment. Thus, the presented test strategy risk assessment considers information on pristine as well as aged ENMs as, based on the durability of the ENMs in the environment, alterations of the physical–chemical characteristics of ENMs are likely to occur and important to consider.

## Conclusion

A test strategy is presented taking nanospecific aspects into account. The strategy for ecotoxicology is already more concrete than for environmental fate and is intended as a starting point for further discussions. There are still several gaps, such as

- Threshold values for the production volume (Fig. 1, step 1)
- Identification of suitable screening tests for substances with production volumes below the threshold value (Fig. 1, step 1)
- Trigger value for the screening tests to differentiate between “significant” and “not significant” effects (Fig. 1, step 1)
- Sensitivity of aquatic tests compared to terrestrial tests; in this context, the research gaps listed for aquatic tests [22] and the spiking methods for terrestrial tests (dry spiking vs. wet spiking) have to be considered (see “Comprehensive testing”)
- Illumination-dependent ecotoxicity of ENMs not specifically designed to feature photocatalytic activity (see “Comprehensive testing”)
- Further information on mode of action of ENMs to improve risk assessment (see “Comprehensive testing”)
- Further information on specific effects currently not included in risk assessment (e.g. immunotoxicity, genotoxicity, multi-generation tests, necessity of further test organisms such as sediment organisms living and grazing on the sediment as well as aquatic macrophytes) (see “Alternative test systems”)
- Current tests for fate endpoints are based on equilibrium situations. For fate testing, the test guidelines have to be modified to address the fact of non-equilibrium situations (e.g. OECD TG 106 adsorption/desorption) (see “PEC assessment, PEC models”).

These gaps have to be filled in the near future to develop the test strategy further. The test strategy features a general approach to test and assess fate and effects of NMs. It features a first attempt to systematically test and assess effects and fate of ENMs in the environment. It has to be noted that the strategy is not yet developed sufficiently specified to fulfil the information requirements of certain legislation (e.g. plant protection act, biocide regulation, REACH). However, the adaption of single elements of the strategy to the specific needs of certain legislation will make a valuable contribution for the adjustment to the testing of nanomaterials.

## Materials

The test strategy has been developed based on published literature, the knowledge of national and international discussions, after comparison with proposals presented by the European Commission and by German Federal Authorities. It also takes into account the conclusions made by the OECD WPMN which held an expert meeting in January 2013 [5].

To select appropriate parameters, test design and test methods for the test strategy on ENMs, recent literature was compiled for ecotoxicology and environmental fate-related key words. The following key words were applied and combined:

Nanoparticles, nanomaterials, ecotoxicology, nano, titanium dioxid, ecotoxicology, silver, soil, terrestrial, aquatic, toxicity, solubility, dissolution, release, partitioning, adsorption, desorption, sorption, sedimentation, transport, mobility, distribution, stability, hydrolysis, degradation, transformation, bioaccumulation, bioavailability, fate, PEC assessment, PEC models, PEC modelling, risk assessment.

The substances silver and titanium dioxide were specifically selected as much ecotoxicological work is done for these two types of nanomaterials.
